# Longitudinal relationship between experience of sexual harassment and 2-year body image and weight outcomes in adolescence: mediation through self-objectification and psychological distress

**DOI:** 10.1007/s40519-022-01432-4

**Published:** 2022-07-14

**Authors:** Samantha Hayes, Alexandra Lonergan, Nora Trompeter, Kay Bussey, Phillipa Hay, Deborah Mitchison

**Affiliations:** 1 School of Psychology, Western Sydney University, 51 Captain Cook Drive, Kurnell, Sydney, NSW 2231 Australia; 2 School of Psychology, Macquarie University, Sydney, NSW Australia; 3 Centre for Emotional Health, Department of Psychology, Macquarie University, Sydney, NSW Australia; 4 Camden and Campbelltown Hospitals, SWSLHD, Campbelltown, NSW Australia; 5 School of Medicine, Translational Health Research Institute, Western Sydney University, Sydney, NSW Australia

**Keywords:** Sexual harassment, Body image, Weight/shape concern, Weight outcomes, BMI

## Abstract

**Background:**

The current study investigated the experience of sexual harassment as a risk factor for weight gain and weight/shape concerns in a community sample of adolescents, with potential mediating factors self-objectification and psychological distress.

**Method:**

1034 Australian adolescents (aged 11 to 19 years) from the EveryBODY longitudinal study of disordered eating pathology participated. Data were collected through online surveys annually for 3 years. Participants completed self-report measures of demographics, sexual harassment, psychological distress, self-objectification, weight/shape concerns and BMI percentile.

**Results:**

A parallel mediation model adjusting for baseline scores found no direct effect between baseline experiences of sexual harassment and change in BMI percentile or weight/shape concern after 2 years. Experiences of sexual harassment significantly increased self-objectification scores after 1 year in female adolescents. Subsequently, higher self-objectification significantly increased the risk of greater weight/shape concern after 1 year in female adolescents. However, no significant mediating relationship was found in the relationship between sexual harassment and weight/shape concern or BMI percentile for either gender. Psychological distress was found to be a clear risk factor for weight/shape concern in both genders after 1 year.

**Conclusions:**

Intervention programmes in schools should focus on developing policies to reduce sexual harassment, self-objectification and distress in adolescents.

**Level of evidence:**

Level IV, longitudinal multiple time series without intervention.

**Supplementary Information:**

The online version contains supplementary material available at 10.1007/s40519-022-01432-4.

## Introduction

Sexual harassment is defined as any sexual behaviour by another person that is deemed unwanted or unwelcome, including jokes, gestures, comments, or physical touching [1, 2]. Early experiences of sexual harassment may be more detrimental as they could directly impact an individual’s perspective of themselves and their environment during a fundamental developmental stage. A recent Australian study found that 41.2% of adolescents reported experiencing at least one form of sexual harassment in the previous 10 weeks [3]. Importantly, this study found no significant gender differences, with 42.5% of boys and 40.0% of girls reporting sexual harassment in the previous school term [3]. Sexual harassment therefore appears to be relevant to both male and female adolescents, highlighting the importance of understanding its long-term consequences.

### The relationship between sexual harassment and body image and weight outcomes

Puberty is a complex time of social and physical changes in body shape and appearance. It can be a time of incongruence between appearance and societal ideals, resulting in greater weight/shape concern, disordered eating pathology and potential weight stigmatisation [4, 5]. Research has also found that adolescents who are at higher weights report disproportionately higher rates of all forms of harassment than other adolescents [6]. Sexual harassment has been found to be associated with self-objectification, self-surveillance, greater weight/shape concern, psychological distress and disordered eating across varying age groups [4, 5]. No research to date has specifically looked at whether sexual harassment predicts weight outcomes long term. It is thus unknown whether increased weight predisposes adolescents to forms of harassment or whether experiences of sexual harassment may predict long-term weight gain.

Exposure to sexual harassment can result in an individual shifting their perspective to be more appearance focused, which can manifest in compulsive behaviours such as checking, reassurance seeking and attempts to fix perceived defects [7]. Similarly, a cross-sectional paper found that sexual harassment was associated with higher levels of weight/shape concern regardless of gender in young adults [8]. A recent meta-analysis found sexual harassment was significantly associated with disordered eating pathology [9]. This review found only one longitudinal study, which was conducted on an early adolescent sample and found that sexual harassment was directly and indirectly related to global disordered eating pathology after 4 years, irrespective of gender [5]. However, this study did not control for baseline levels of eating pathology, so was unable to examine whether sexual harassment was causally related to *increases* in disordered eating over time. Although research has demonstrated a cross-sectional relationship between sexual harassment and disordered eating broadly, there has been no prospective investigation to date [8, 10]. Thus, the current paper aims to address this gap, elucidating whether sexual harassment is indeed a causal risk factor for disordered eating pathology among adolescents.

### Sexual harassment, sexual abuse and their differing relationship with disordered eating

Sexual abuse is defined as any unwanted sexual activity where there is force, threats and/or no consent [11]. The majority of research concludes that while there is an association between disordered eating and sexual abuse, it is not specific to eating disorders, rather such traumatic experiences function as a general risk factor for any psychological disorders [12]. In comparison to the literature on abuse, there have been minimal studies that have specifically investigated associations between sexual harassment and disordered eating pathology [10, 12]. However, due to the body-focused evaluations and consequences of these experiences, it has been suggested that sexual harassment may act as a more disorder-specific risk factor, promoting the development of disordered eating [3, 13]. It is agreed consistently that early experiences of sexual harassment and/or sexual abuse can have negative long-term consequences that can progress into adulthood [14]. Importantly, previous research has highlighted that posttraumatic stress symptoms can moderate the relationship between childhood sexual abuse and disordered eating in an adult sample and thus the importance of considering mediating factors in the relationship between sexual harassment and disordered eating in the current study [15, 16].

### Self-objectification as a mediator of this relationship

Objectification theory was developed as a feminist sociocultural framework to explain how everyday objectifying experiences lead women to view themselves from an outsider’s perspective [7]. In this context, women are socialised to view and treat themselves as an “object” and valued predominantly for their appearance, use or consumption by others [7]. Objectification theory has contributed to current understandings of the impact of sexual harassment on disordered eating pathology and body image concerns [7], with self-objectification, alongside psychological distress and body shame, found to be a significant mediator of the relationship between sexual harassment and disordered eating pathology [9]. This appearance-focused shift can result in striving towards unrealistic sociocultural appearance ideals (e.g. lean and toned), which can subsequently result in greater experiences of objectificaton from others, perpetuating this unrelenting cycle [4, 5]. Thus, further investigation the role of self-objectication as a mediator of sexual harassment outcomes is needed to fully understand the consequences of these experiences.

### Psychological distress as a mediator

Previous research has demonstrated that individual psychopathology, psychological distress and anxiety were significant *concurrent* mediators of sexual harassment and disordered eating [10, 13, 17]. Experiences of sexual harassment may increase baseline levels of distress which can subsequently result in disordered eating behaviour as a means to self-soothe or overcompensate [4, 5]. Previous research has also found that posttraumatic stress disorder (PTSD) symptoms; which are often a result of sexual harassment and other adverse experiences, are also associated with greater disordered eating behaviours and higher rates of utilising food for comfort [16]. It has also highlighted that the combination of sexual violence and disordered eating is associated with severe psychological consquences [14].Thus, further investigation into psychological distress as an additional mediator of the relationship between sexual harassment and its outcomes is required to fully understand whether predictive links can be identified and thus considered in future interventions.

### Study aims

Experiences of sexual harassment have become common within society thus increasing the impact of their consequences [12]. Research consistently supports the concept that sexual harassment is directly associated with disordered eating pathology and that self-objectification and psychological distress have a concurrent role in this relationship [9]. However, no study to date has specifically investigated the contributing relationship of sexual harassment on weight outcomes, and weight/shape concern in adolescent population, nor potential mediators of this relationship. The aim of the current study is to further investigate the experience of sexual harassment and long-term disordered eating outcomes in an adolescent population, by utilising weight/shape concern and BMI percentile as outcome variables. We also aimed to examine the mediating roles of self-objectification and psychological distress on this relationship. Based on previous findings, we hypothesise:

1. A direct relationship between the experience of sexual harassment at baseline and increased BMI percentile after 2 years, relative to adolescents with no experienced sexual harassment at baseline, controlling for baseline BMI.
2. A direct relationship between the experience of sexual harassment at baseline and increased weight/shape concern after 2 years, compared to adolescents with no experienced sexual harassment at baseline, controlling for baseline measures of weight/shape concern.
3. The experience of sexual harassment at baseline will lead to greater increases in self-objectification and psychological distress after 1 year, which will subsequently mediate the relationship between baseline sexual harassment and increases in weight/shape concern and BMI percentile after 2 years, controlling for baseline measures of covariates.

### Method

#### Participants

The current study used data from the EveryBODY Study, an Australian longitudinal investigation of body image concerns and disordered eating in an adolescent population [*full details:* 18]. Briefly, baseline data was collected from students at 13 secondary schools (36% of approached schools) across the Sydney, Newcastle and Hunter Regions of New South Wales; five schools opted out of follow-up surveys, leaving eight schools comprising the longitudinal cohort used in the current study. Participants who had left school by the time of either of the follow-up waves and who had given permission to be contacted were emailed an invitation to participate in the follow-up online surveys. These participants had the choice to receive a $10 gift card or to go into a prize draw for 1 of 10 $100 gift cards. To create a true longitudinal analysis only participants who completed all three waves were included and therefore a total of 1056 students participated in all three survey waves (Wave 1 = baseline, Wave 2 = 1-year follow-up, Wave 3 = 2-year follow-up). Of these, 12 were excluded due to non-serious responses, 8 for excessive missing data (greater than 90% missing) and 2 withdrew consent. Final analyses were conducted on 1034 adolescents (57.4% girls), between the age of 12 years 0 months and 20 years 5 months over 3 years. Within this sample, 87.6% of students reported being born in Australia, 7.1% in Asia, 2.8% in Europe, 1.4% in Oceania/Pacific (other than Australia), 0.6% in Africa, 0.5% in North America and 0.1% did not specify their country of birth. Participants in the current study were younger, *t*(1794.42) = 21.73, *p* = 0.000, Cohen’s *d* = 0.92 (large effect), and had a lower BMI percentile, *t*(1927) = 3.429, *p* = 0.001, Cohen’s *d* = 0.16 (small effect), compared to those who only participated at Wave 1. Similarly, current participants were also younger, *t*(1393.68) = 4.887, *p* = 0.000, Cohen’s *d* = 0.23 (small effect), had a lower BMI percentile, *t*(1753) = 2.42, *p* = 0.02, Cohen’s *d* = 0.12 (small effect) and were less ethnically diverse, *t*(1393.81) = 3.23, *p* = 0.001, Cohen’s *d* = 0.15 (small effect), than participants who withdrew after Wave 2. No differences were found between included and excluded participants based on gender.

### Procedure

Ethics approval was received from the university ethics committee, the Catholic Education Office, and the New South Wales Department of Education. The online survey consisted of questions regarding demographics, disordered eating pathology, weight/shape concerns, psychological distress, self-objectification and sexual harassment. A passive parental consent procedure was utilised, whereby information was sent out to parents and consent was assumed unless parents actively opted their child out of the study. Only assenting students completed the online survey during a 50-min class under teacher supervision. Pilot testing was conducted on a small group of adolescents prior. At the completion of the study, adolescents were provided with resources and local referral pathways for disordered eating and general mental health. They were also offered the opportunity to enter a prize draw to win one of ten $100 vouchers.

### Measures

#### Demographic information

Participants were asked to complete questions on their age, biological sex, country of birth, parents’ country of birth, parents’ education, their school grade, sexuality and their postcode (later converted to a socioeconomic index for area—SEIFA—score). SEIFA scores are created using information about households in a particular area to determine socioeconomic status (SES). Previous studies have highlighted the great diversity of this sample population by utilising SEIFA scores [3]. SES has been assessed utilising this population prior and no significant difference was found between male and female participants and therefore was not was included in this current study [3].

#### Sexual harassment

The sexual harassment survey involved a nine-item modified version [19] originally developed a 10-item survey by removing 5 items from the 14-item [1] sexual harassment survey and adding an extra item as recommended by the Board of Education (i.e. commenting on or ranking your private parts). The current study removed the least endorsed item (wrote sexual messages/graffiti about you on bathroom walls, in locker rooms, etc.) [19]. Participants were required to rate how often the listed behaviours were experienced in an unwanted manner in the last school term (roughly 10 weeks) on a five-point scale (0 = *never* to 4 = *daily*). Total scores of SH were dichotomised into no experience of SH (total score = 0) or any experience of SH (total score > 0). This modified version demonstrated good reliability within an adolescent sample for both genders [19]. The current study found good internal consistency with Cronbach’s α for boys: Wave 1 = 0.87, Wave 2 = 0.88, Wave 3 = 0.90, and for girls: Wave 1 = 0.84, Wave 2 = 0.89, Wave 3 = 0.90.

#### Self-objectification

The Self-Objectification Questionnaire [SOQ; 20] was utilised to indicate the extent to which individual’s view their bodies. Participants are required to rank ten body attributes in order of importance to them (1 = *least important*; 10 = *most important*). Five attributes were appearance related (weight, sex appeal, physical attractiveness, firm/sculpted muscles and measurements), while the other five were competence related (energy, health, strength, fitness and physical coordination). Final scores were obtained by calculating the difference between the sum of competence scores and the sum of appearance scores; possible scores range from − 25 to 25. Higher scores indicated higher levels of self-objectification. Due to scores being ranked, internal consistency could not be calculated. Construct validity has been previously demonstrated by moderate positive correlations with the Appearance Anxiety Questionnaire and the Body Image Assessment [20] and has been frequently used in other adolescent population studies to measure self-objectification [21].

#### Psychological distress

The Kessler Psychological Distress Scale [K10; 22] is a ten-item measure of psychological distress over the past 4 weeks. Items were rated on a five-point scale (1 = *none of the time* to 5 = *all of the time*); higher scores indicate higher levels of distress. The use of K10 has been previously validated, demonstrated good reliability and highlighted unidimensional within an Australian child and adolescent population [23]. Excellent internal consistency was shown in the current study with Cronbach’s α for boys: Wave 1 = 0.90, Wave 2 = 0.91, Wave 3 = 0.92, and for girls: Wave 1 & 2 = 0.94, Wave 3 = 0.95.

#### Weight/shape concern

Weight/shape concern was measured using the 12 items of the weight and shape concern subscales of the Eating Disorders Examination Questionnaire [EDE-Q, 24] which were combined to assess the frequency/severity of a participant’s weight and shape concerns over the past 4 weeks. Questions were rated using a 7-point scale with higher scores representing greater concern (0 = *No days/Not at all* to 6 = *Everyday/Markedly*). This combined use of the weight/shape concern subscales has been previously validated and demonstrated good reliability in an Australian adolescent sample [25]. The current study found excellent internal consistency with Cronbach’s α for boys: Wave 1 =  0.93, Wave 2 and 3 =  0.94, and for girls: Wave 1, 2 and 3 =  0.96.

#### Body mass index (BMI) percentile

Participants were asked to self-report their height (cm) and weight (kg), with these measurements then converted to age- and gender-adjusted BMI percentiles based on the CDC Guidelines [26]. BMI percentiles have been found to be a better representation of anthropometric measurements in adolescents than unadjusted BMI [27]. BMI percentile is used in the current study as a measure of long-term weight outcomes.

### Statistical analysis

#### Multiple imputation

Most variables had some level of incomplete data (0.2% to 13.2%) due to students not reaching the end of the survey. Using Little’s MCAR test it was determined that data was missing completely at random and then missing data was replaced by imputed values. Multiple imputation was utilised with linear regression employed for scale variables. The following were entered as both dependent and predictor variables: sexual harassment total score, self-objectification total score, psychological distress total, height, weight and combined weight/shape concern. Baseline measures of mediating and dependent variables were controlled for as covariates. Five imputations were computed and the pooled results are reported.

#### Conditional process analysis

**Fig. 1:**
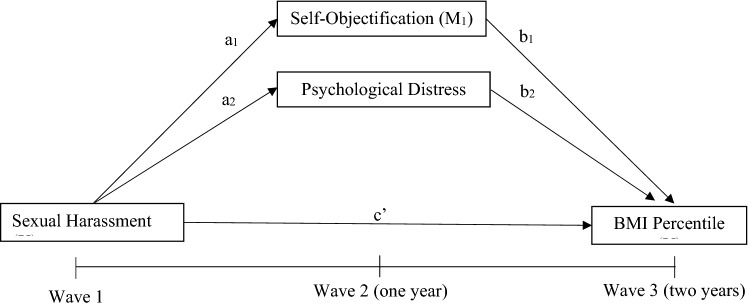
Model **1—**simplified parallel mediation model for sexual harassment and BMI percentile. Age at Wave 1 was utilised as a covariate, while baseline adjustments of each measure were also entered into the model. For clarity, covariate pathways are not shown

**Fig. 2:**
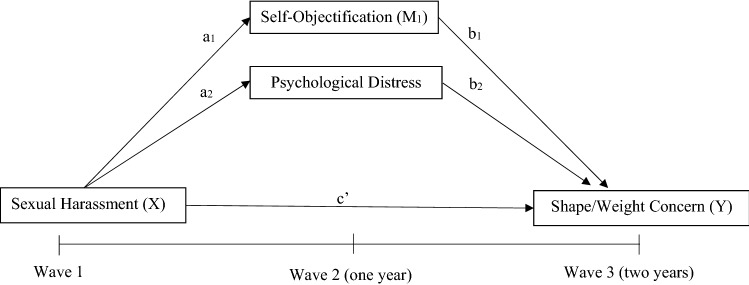
Model **2—**simplified parallel mediation model for sexual harassment and weight/shape concern. Age and BMI percentile at Wave 1 was utilised as a covariate, while baseline adjustments of each measure were also entered into the model. For clarity, covariate pathways are not shown

A parallel mediation model was utilised to investigate whether the relationship between sexual harassment at baseline and change in BMI percentile or weight/shape concern after 2 years was mediated by change in psychological distress and self-objectification after 1 year. This process measures the relationship between an independent variable and each mediating variable while simultaneously using them as predictors of an outcome variable. All analyses were split by sex due to baseline differences. In the first model, Wave 3 BMI percentile was entered as the outcome variable, Wave 1 sexual harassment as the predictor variable and Wave 2 psychological distress and self-objectification constructs as mediators as displayed in Fig. 1. Due to differences in baseline demographics and the association between age and weight status, this model also controlled for Wave 1 age [28]. The second model utilised the same predictor and mediating variables; however, Wave 3 weight/shape concern was entered as the outcome variable, as represented in Fig. 2. Due to associations, baseline levels of age and weight status were controlled for [29]. To facilitate testing of these relationships, model 1 was adjusted for baseline levels of BMI percentile, self-objectification and psychological distress, while model 2 was adjusted for baseline levels of weight/shape concern, self-objectification and psychological distress.

#### Data analysis

Independent *t* tests were used to compare differences in the study variables between male and female adolescents. All hypotheses were tested using a condition process modelling programme, PROCESS, to test for both direct and indirect effects, employing PROCESS Model 4, parallel mediation [30]. All indirect effects were subjected to follow-up bootstrap analyses with 10,000 bootstrap samples and 95% bias-corrected confidence intervals.

### Results

#### Sex differences

Table 1 displays the descriptive statistics and independent *t* test scores. At baseline, sexual harassment was experienced at least once in the past 10 weeks by 42.8% of male adolescents and 36.7% of female adolescents. Females displayed higher psychological distress and greater weight/shape concern at all three waves. Compared to females, males were significantly older and reported significantly more sexual harassment across the 3 years and a higher BMI percentile and greater self-objectification at Wave 2 and 3. These differences justify the approach of splitting subsequent analyses by sex.

**Table 1:** Characteristics of male and female participants

	Male (*n* = 441)		Female (*n* = 593)		Df	T	*p*	Cohen’s *d*
	M	SD	M	SD				
Wave 1								
Age	13.87	1.19	13.7	1.22	1032	**2.23**	0.026	0.14
Ethnicity	1.34	0.97	1.26	0.92	922.49	1.28	0.200	0.08
SH	0.43	0.50	0.37	0.48	934.19	1.97	0.055	0.12
SOQ	8.64	12.06	7.31	12.41	1032	1.72	0.089	0.11
K10	16.73	6.95	21.14	9.87	1029.08	**− 8.42**	0.000	0.52
BMI	51.38	32.15	49.04	30.59	1032	1.19	0.241	0.08
SWC	0.84	1.24	1.96	1.81	1025.12	**− 11.89**	0.000	0.72
Wave 2								
Age	14.91	1.21	14.67	1.26	1032	**3.10**	0.002	0.19
SH	0.45	0.50	0.38	0.49	935.46	**2.28**	0.033	0.14
SOQ	10.77	12.11	8.65	12.44	1032	**2.75**	0.006	0.17
K10	17.78	8.02	22.63	10.19	1028.66	**− 8.56**	0.000	0.53
BMI	54.10	32.13	49.84	28.90	889.52	**2.20**	0.028	0.14
SWC	0.91	1.28	2.28	1.84	1027.58	**− 14.22**	0.000	0.86
Wave 3								
Age	15.78	1.23	15.62	1.25	1032	**2.03**	0.043	0.13
SH	0.45	0.50	0.38	0.49	934.57	**2.23**	0.028	0.14
SOQ	9.85	12.08	8.05	13.32	992.34	**2.27**	0.028	0.14
K10	18.46	8.57	24.17	10.21	1018.60	**− 9.78**	0.000	0.61
BMI	55.59	31.38	51.56	28.36	892.16	**2.14**	0.033	0.14
SWC	0.92	1.28	2.44	1.83	1027.93	**− 15.69**	0.000	0.96

Bold values indicate better results than other filtering methods

*SH* sexual harassment, *SOQ* self-objectification, *K10* psychological distress, *BMI* body mass index percentile, *SWC* weight/shape concern

#### Correlations

Pearson correlation analyses are displayed in Table 2. Significant large correlations between BMI percentiles across time for both genders: Wave 1 and Wave 2 (Girls = 0.55, Boys =  0.64), Wave 1 and Wave 3 (Girls = 0.54, Boys = 0.60), and Wave 2 and Wave 3 (girls =  0.66, boys =  0.68). Large correlations were also found between levels of weight/shape concern across each wave for both genders: Wave 1 and Wave 2 (girls = 0.61, boys =  0.67), Wave 1 and Wave 3 (girls = 0.54, boys = 0.57), and Wave 2 and Wave 3 (girls = 0.69, boys =  0.71). These correlations are indicative of a high level of stability in the constructs over time; thus, both adjusted and unadjusted models for baseline levels of outcome measures are presented to highlight longitudinal associations (unadjusted models) and potential predictive pathways (adjusted models). Reporting unadjusted findings also facilitates comparisons between previous unadjusted research.

**Table 2:** Correlations for both male and female adolescents

		1		2		3		4	5	6		7		8		9		10	11		12		13		14		15			16	17	
Wave 1																																
1	Age		− 0.02		0.18***		− 0.17***		0.20***		0.15***		0.23***		0.14**		− 0.13**	0.13**		0.12**		0.16***		0.05		− 0.09*		0.11**	0.10*			0.13***
2	Ethnicity	0.003			0.002		− 0.01		− 0.02		− 0.01		− 0.06		− 0.05		0.06	− 0.03		0.02		− 0.06		− 0.02		0.04		− 0.03	− 0.003			− 0.08
3	SH	0.02	0.01				− 0.21***		0.28***		0.07		0.27***		0.38***		− 0.19***	0.20***		0.08		0.16***		0.26***		− 0.14**		0.19***	0.02			0.17***
4	SOQ	− 0.04	− 0.07		− 0.13**				− 0.28***		− 0.08		− 0.34***		− 0.11*		0.38***	− 0.24***		− 0.02		− 0.27***		− 0.09*		0.33***		− 0.21***	− 0.04			− 0.21***
5	K10	0.04	0.01		0.19***		− 0.21***				0.19***		0.60***		0.16***		− 0.23***	0.61***		0.17***		0.41***		0.20***		− 0.23***		0.53***	0.17***			0.38***
6	BMI	0.17***	0.03		0.04		− 0.07		0.14***				0.29***		0.06		− 0.09*	0.12**		0.55***		0.27***		0.000		− 0.07		0.14***	0.54***			0.27***
7	SWC	0.11*	0.10*		0.22***		− 0.24***		0.49***		0.27***				0.18***		− 0.25***	0.42***		0.30***		0.61***		0.18***		− 0.27***		0.38***	0.31***			0.54***
Wave 2																																
8	SH	0.06	− 0.02		0.28***		0.02		0.17***		0.04		0.12*				− 0.19***	0.25***		0.07		0.22***		0.32***		− 0.13**		0.16***	0.04			0.17***
9	SOQ	.− 0.03	− 0.04		− 0.07		0.31***		− 0.17***		− 0.05		− 0.24***		− 0.15**			− 0.31***		− 0.03		− 0.43***		− 0.18***		0.48***		− 0.26***	− 0.07			− 0.32***
10	K10	0.14**	− 0.01		0.08		− 0.13**		0.53***		0.17***		0.32***		0.25***		− 0.26***			0.14***		0.59***		0.22***		− 0.28		0.69***	0.14***			0.43***
11	BMI	0.12*	0.06		0.06		− 0.06		0.07		0.64***		0.23***		.0.02		− 0.02	0.09				0.31***		0.01		− 0.02		0.19***	0.66***			0.30***
12	SWC	0.14**	0.12**		0.09		− 0.17***		0.39***		0.30***		0.67***		0.14**		− 0.28***	0.55***		0.26***				0.16***		− 0.36***		0.45***	0.35***			0.69***
Wave 3																																
13	SH	0.05	0.02		0.18**		− 0.20***		0.17***		0.000		0.17**		0.25***		− 0.15**	0.14**		0.02		0.14**				− 0.16***		0.24***	− 0.03			0.19***
14	SOQ	.− 0.02	− 0.04		− 0.04		0.34***		− 0.16***		0.002		− 0.20***		− 0.09		0.39***	− 0.22***		− 0.03		− 0.25***		− 0.15**				− 0.35***	− 0.03			− 0.41***
15	K10	0.10*	0.10*		0.05		− 0.14***		0.47***		0.15***		0.33***		0.10*		− 0.19***	0.57***		0.15**		0.51***		0.22***		− 0.23***			0.19***			0.54***
16	BMI	0.08	0.06		0.06		− 0.08		0.07		0.60***		0.25***		− 0.03		− 0.02	0.07		0.68***		0.27***		0.04		− 0.08		0.13**				0.37***
17	SWC	0.14**	0.11*		0.10*		− 0.15***		0.33***		0.30***		0.57***		0.10*		− 0.24***	0.39***		0.29***		0.71***		0.16**		− 0.32***		0.54***	0.33***			

Correlations above the diagonal are for girls. Correlations below the diagonal are for boys

*SH* sexual harassment, *SOQ* self− objectification, *K10* psychological distress, *BMI* body mass index percentile, *SWC* weight/shape concern

*p ≤ 0.05, **p ≤ 0.01,***p ≤ 0.001

#### BMI percentile

**Fig. 3:**
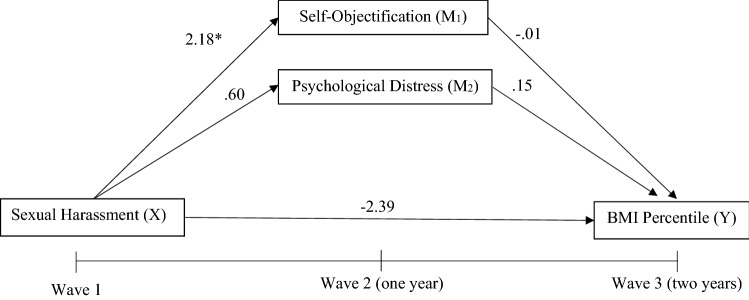
Adjusted parallel mediation model for sexual harassment and BMI percentile in female adolescents. Age, BMI percentile, self-objectification and psychological distress at Wave 1 were all controlled for. *p ≤ .05, **p ≤ .01, ***p ≤ .001

**Fig. 4:**
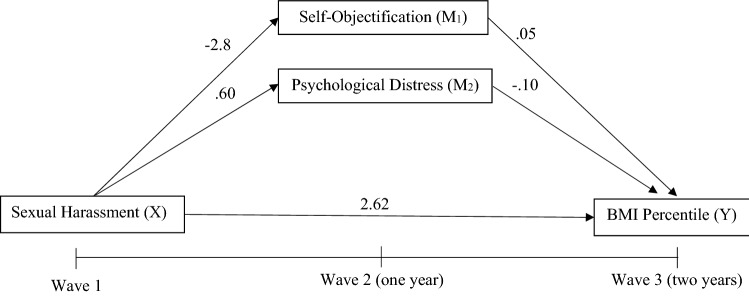
Adjusted parallel mediation model for sexual harassment and BMI percentile in male adolescents. Age, BMI percentile, self-objectification and psychological distress at Wave 1 were all controlled for. *p ≤ .05, **p ≤ .01, ***p ≤ .001

Hypothesis 1 was not supported as the adjusted parallel mediation analysis found no direct effect between sexual harassment at Wave 1 and BMI percentile at Wave 3 for either gender. When considering the prediction of change over time (i.e. adjusting for baseline levels of the outcome and mediating variables), there was no direct relationship found between Wave 1 reporting of sexual harassment and change in BMI percentile after 2 years. A significant relationship was, however, found with reported experiences of sexual harassment at Wave 1 predicting greater increases in self− objectification after 1 year in Wave 2 for female adolescents (a_1_ =  − 2.18, *p* = 0.036). No other hypothesised predictive relationships were observed in the adjusted models; however, higher BMI percentile (for both males and females) and greater levels of psychological distress (for females only) at Wave 1 predicted greater increases in BMI percentile at Wave 3; see Table 3. See Figs. 3 and 4 for mediation model for female and male adolescents, respectively.

**Table 3:** Adjusted parallel mediation models with BMI percentile as outcome for both male and female adolescents

	Females			Males		
	B	SE	95%CI	B	SE	95%CI
BMI percentile W3						
Age W1	0.30	0.84	− 1.35, 1.96	− 0.56	1.02	− 2.57, 1.45
BMI percentile W1	0.48***	0.03	0.42, 0.55	0.59***	0.04	0.51, 0.66
Self− objectification W1	0.05	0.08	− 0.12, 0.21	− 0.11	0.10	− 0.31, 0.09
Psychological distress W1	0.23*	0.11	0.02, 0.44	− 0.14	0.18	− 0.49, 0.22
Direct effect of sexual harassment W1 (*X*) on BMI percentile W3 (*Y*)	− 2.39	2.17	− 6.66, 1.87	2.62	2.48	− 2.26, 7.50
Path from sexual harassment W1 (*X*) to self− objectification W2 (*M*_1_)	2.18*	1.02	− 4.19, − 0.16	− 0.28	1.13	− 2.51, 1.95
Path from sexual harassment W1 (*X*) to Psychological distress W2 (*M*_2_)	0.60	0.73	− 0.83, 2.03	− 0.36	0.66	− 1.66, 0.94
Path from self-objectification W2 (*M*_1_) to BMI percentile W3 (*Y*)	− 0.01	0.09	− 0.19, 0.16	0.05	0.11	− 0.16, 0.26
Path from psychological distress W2 (*M*_2_) to BMI percentile W3 (*Y*)	0.15	0.12	− 0.09, 0.40	− 0.10	0.18	− 0.46, 0.26

	Females			Males		
	B	SE	95%CI	B	SE	95%CI
Mediation						
Indirect effect of sexual harassment W1 (*X*) on BMI percentile W3 (*Y*) through self-objectification W2 (*M*_1_) and psychological distress W2 (*M*_2_)	− 2.27	2.16	− 6.52, 1.97	2.65	2.48	− 2.21, 7.52
Indirect effect of sexual harassment W1 (*X*) on BMI percentile W3 (*Y*) through self-objectification W2 (*M*_1_)	0.03	0.22	− 0.42, 0.49	− 0.01	0.15	− 0.35, 0.31
Indirect effect of sexual harassment W1 (*X*) on BMI percentile W3 (*Y*) through psychological distress W2 (*M*_2_)	0.09	0.17	− 0.21, 0.51	0.05	0.18	− 0.27. 0.51
	*R*^*2*^ = 0.29			*R*^*2*^ = 0.36		
	*F*(5, 587) = 48.61, *p* < 0.001			*F*(5, 435) = 49.87, *p* < 0.001		

Bold values indicate better results than other filtering methods

*W1* wave 1, *W2* wave 2, *W3* wave 3

*X* predictor variable, *Y* dependent variable, *M*_*1*_*,* *M*_*2*_ and *M*_*3*_ mediating variables

Unstandardised coefficients from bootstrapped analyses are presented

*p ≤ 0.05, **p ≤ 0.01, ***p ≤ 0.001

^a^CI did not include zero, indicating a significant effect

#### Weight/shape concern

**Fig. 5:**
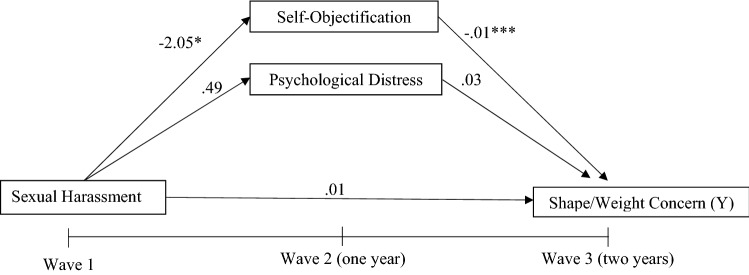
Adjusted parallel mediation model for sexual harassment and weight/shape concern in female adolescents. Age, BMI percentile, weight/shape concern, self-objectification and psychological distress at Wave 1 were all controlled for. * *p* ≤ .05, **p ≤ .01, ***p ≤ .001

**Fig. 6:**
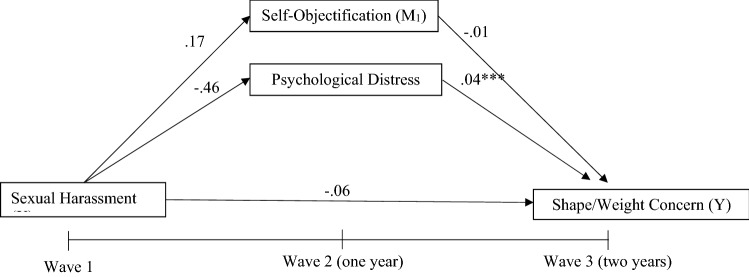
Adjusted parallel mediation model for sexual harassment and weight/shape concern in male adolescents. Age, BMI percentile, weight/shape concern, self-objectification and psychological distress at Wave 1 were all controlled for. *p ≤ .05, **p ≤ .01, ***p ≤ .001

Hypothesis 2 was also not supported, as the adjusted parallel mediation analysis found no direct effect between sexual harassment at Wave 1 and weight/shape concern at Wave 3 for either gender. Adjusting for change in the outcome and mediators over time, no direct relationship was found between Wave 1 sexual harassment and changes in weight/shape concern after 2 years, in either gender. However, significant relationships were found among adolescent females who experienced sexual harassment at Wave 1 and greater increases in self-objectification by Wave 2 (*a*_1_ =  − 2.05, *p* = 0.050); also, greater self-objectification at Wave 2 predicted greater increases in weight/shape concern by Wave 3 (*b*_1_ =  − 0.01, *p* = 0.000). Another significant relationship was found among adolescent females who reported higher levels of psychological distress at Wave 2 predicting greater increases in weight/shape concern by Wave 3 (*b*_2_ = 0.03, *p* = 0.000). However, the adjusted model did not find a significant mediation with the relationship between sexual harassment and weight/shape concern through self-objectification. The relationship between experience of sexual harassment at Wave 1 and higher score of psychological distress at Wave 2 lost its significance when baseline scores were adjusted for. For male adolescents, a positive relationship was observed between higher psychological distress at Wave 2 which predicted greater increases in weight/shape concern by Wave 3 (*b*_2_ = 0.04, *p* = 0.000). Other significant relationships observed in the adjusted models included Wave 1 weight/shape concern and BMI percentile for both males and females, and Wave 1 psychological distress for females predicting greater increases in weight/shape concern by Wave 3. See Table 4 Figs. 5 and 6 for adjusted mediation model for female and male adolescents, respectively.

**Table 4:** Parallel mediation model with weight/shape concern as outcome for both male and female adolescents

	Females			Males		
	B	SE	95%CI	B	SE	95%CI
Weight/shape concern						
Age W1	− 0.02	0.05	− 0.13, 0.09	0.06	0.04	− 0.02, 0.14
BMI percentile W1	0.01*	0.002	0.003,0.01	0.01*	0.002	0.003, 0.01
weight/shape concern W1	0.44*	0.05	0.35, 0.53	0.52*	0.05	0.42, 0.61
Self-objectification W1	− 0.003	0.006	− 0.01, 0.01	− 0.001	0.004	− 0.01, 0.01
Psychological distress W1	0.02***	0.01	0.001, 0.03	0.01	0.01	− 0.01, 0.03
Direct effect of sexual harassment W1 (*X*) on weight/shape concern W3 (*Y*)	0.01	0.13	− 0.25, 0.27	− 0.06	0.10	− 0.26, 0.13
Path from sexual harassment W1 (*X*) to self-objectification W2 (*M*_1_)	− 2.05***	**1.03**	− 4.07, − 0.03	0.17	1.13	− 1.79, 2.15
Path from sexual harassment W1 (*X*) to psychological distress W2 (*M*_2_)	0.49	0.73	− 0.94, 1.93	− 0.46	0.67	− 1.77, 0.85
Path from self-objectification W2 (*M*_1_) to weight/shape concern W3 (*Y*)	− 0.01*	0.01	− 0.212, 0.002	− 0.01	0.001	− 0.02, 0.001
Path from psychological distress W2 (*M*_2_) to weight/shape concern W3 (*Y*)	0.03*	0.01	0.02, 0.04	0.04*	0.01	0.02, 0.05

	Females			Males		
	B	SE	95%CI	B	SE	95%CI
Mediation						
Indirect effect of sexual harassment W1 (*X*) on weight/shape concern W3 (*Y*) through self-objectification W2 (*M*_1_) and psychological distress W2 (*M*_2_)	0.08	0.14	− 0.19, 0.36	− 0.08	0.10	− 0.28, 0.12
Indirect effect of sexual harassment W1 (*X*) on weight/shape concern W3 (*Y*) through self-objectification W2 (*M*_1_)	0.05	0.03	− 0.001, 0.11	− 0.02	0.03	− 0.08, 0.03
Indirect effect of sexual harassment W1 (*X*) on weight/shape concern W3 (*Y*) through psychological distress W2 (*M*_2_)	0.02	0.03	− 0.04, 0.09	− 0.001	0.01	− 0.02, 0.02
	*R*^*2*^ = 0.31			*R*^*2*^ = 0.36		
	*F*(6, 586) = 43.25, *p* < 0.001			*F*(6, 434) = 40.27, *p* < 0.001		

Bold values indicate better results than other filtering methods

*W1* Wave 1, *W2* Wave 2, *W3* Wave 3

*X* predictor variable, *Y* dependent variable *M*_*1*_*,* and *M*_*2*_ mediating variables

Unstandardised coefficients from bootstrapped analyses are presented

*p ≤ 0.05, **p ≤ 0.01, ***p ≤ 0.001

^a^CI did not include zero, indicating a significant effect

Therefore, Hypothesis 3 was also not supported as no significant mediating relationships were observed among female or male adolescents in the relationship between experiences of sexual harassment at Wave 1 and BMI percentile at Wave 3 or weight/shape concern at Wave 3.

## Discussion

The current study aimed to investigate the prospective relationships between sexual harassment and weight/shape concern, and weight outcomes in a representative sample of Australian adolescents. It examined the longitudinal effects of sexual harassment on weight outcomes and weight/shape concern, and whether this relationship was mediated by self-objectification and/or psychological distress. Hypotheses 1 and 2 were not supported, as no direct relationships were identified between baseline sexual harassment and changes in weight outcome, or weight/shape concern after 2 years for either gender. Hypothesis 3 was also not supported; simply, no significant mediating relationships were found for any of the mediators between sexual harassment at baseline and BMI percentile or weight/shape concern after 2 years for either gender. However, a significant relationship was identified between experiences of sexual harassment and higher self-objectification scores after one year in females’ adolescents only. Higher self-objectification scores also increased the risk of greater weight/shape concern after one year in female adolescents. Higher psychological distress was also highlighted as a clear risk factor across both genders on weight/shape concern after 1 year.

Alternatively, self-objectification was positively related to sexual harassment and increased weight/shape concern in female adolescents. These findings suggest that self-objectification may only be positively linked for weight/shape concern among females. These findings were similar to previous research which found self-objectification to be a significant concurrent mediator of the relationship between sexual harassment and disordered eating patterns, in both genders [5, 31]. Only long-term associations were demonstrated for psychological distress (*see* supplementary file), which mediated the relationship between sexual harassment and higher BMI percentile and greater weight/shape concern for female adolescents, but no direct links were identified. Current findings are the first to identify a significant longitudinal link between sexual harassment, self-objectification and weight/shape concern in female adolescents, controlling for baseline measures.

A significant relationship has also been suggested between higher psychological distress and greater weight/shape concern in both genders after 1 year. Psychological distress did not significantly contribute to the mediation of the relationship between sexual harassment and increased BMI percentile or weight/shape concern. Congruently, these findings support previous research that found that psychological distress is a significant risk factor for disordered eating pathology [10, 13].

Overall, no significant mediating relationships were found among male adolescents between sexual harassment and higher BMI percentile or greater weight/shape concern. It was found that development of increased self-objectification and/or psychological distress, may subsequently lead to increases in weight/shape concerns long term; however, this may not be contributed to by sexual harassment. Although, this finding was not associated with the predicting variable, the findings of distress and self-objectification increasing the risk of problems with weight/shape concern are concerning; given that weight/shape concern is associated with a range of deleterious outcomes including disordered eating pathology, substance use and self-harm [32, 33].

Finding no direct relationship between sexual harassment and future weight outcomes and weight/shape concern was inconsistent with previous research, which may be due to the longitudinal nature of the current paper and/or due to adolescence being a time of marked psychosocial development. Previous research has consistently found support that sexual harassment has a direct relationship with disordered eating pathology [9]. However, no study as far as we are aware to date had examined causal relationships in a longitudinal sample that also controls for baseline measures. Indeed, when examining the concurrent correlations in our study within each time wave, we found that sexual harassment was related to psychological distress, self-objectification and weight/shape concern, albeit not BMI percentile. Further, the current study used two new outcome variables of disordered eating pathology that have not been investigated to date and therefore the current study provides a broader understanding of this relationship. Consequently, the current study found that any experience of sexual harassment may not directly increase weight gain or the risk of weight/shape concern in the long term for an adolescent sample.

Consistent support for concurrent associations between sexual harassment and broad disordered eating pathology were identified, with self-surveillance/self-objectification, psychological distress and body shame all suggested to act as mediators on this relationship [*see supplementary file,* 5, 8–9]. The current study extended this research by investigating whether this relationship and its mediators are also found between sexual harassment and weight outcomes, and weight/shape concern in adolescents. The current study supports the finding that self-objectification potentially mediates the relationships between sexual harassment and weight/shape concern, positively linked among females. These results perpetuate support for the objectification theory that experiences of sexual harassment may alter one’s perception of themselves to be more appearance focused, possibly resulting in greater attempts to cope or meet societal expectations through disordered eating pathology [5, 13].

Interestingly, the current study found that across Wave 2 and 3, males reported significantly higher rates of sexual harassment compared to females. This is consistent with some previous research [10] however contradicts others [3]. These current findings however highlight that gender differences should be considered for the development of longitudinal risk factors/outcomes. The lack of findings to support a direct relationship between sexual harassment and BMI percentile or weight/shape concern may be a reflection that not all individuals who experience sexual harassment will subsequently experience increases in body mass or weight/shape concern and/or that these outcomes may only occur under certain circumstances: e.g. being female and experiencing high levels of self-objectification. It may also be that previous findings are only present for disordered eating behaviours; such as binge eating, purging, or fasting. To date, this is the first longitudinal study to utilise BMI percentile and weight/shape concern as outcome measures for the negative effects of sexual harassment.

### Clinical implications

The current study has provided a greater foundation to understand the role of sexual harassment on different long-term outcomes for an adolescent population; most notably the direct relationship between sexual harassment and long-term increases in self-objectification among adolescent girls. Given the current findings and the supporting literature, it would suggest that adolescents should also be assessed for experiences of sexual harassment and other adverse life experiences due to the implications of these experiences and comorbid symptoms, (e.g. psychological distress). Increased self-objectification can exacerbate thin ideals, thus increasing the development of body image related behaviours; such as checking, reassurance seeking, which long term may develop into unhealthy eating and weight control behaviours; such as, binge eating, purging, or fasting [4, 5]. Sexual harassment is still highly prevalent in high schools [1, 3] and therefore more emphasis should be placed on developing preventative programmes. School programs have a tendency to focus on bullying or sexual abuse without emphasising sexual harassment and its consequences specifically [5]. Programmes need to focus on reducing sexual harassment through teaching appropriate and respectful interactions between peers and de-stigmatising speaking up about inappropriate experiences. Given findings in the current study, including strategies to reduce self-objectification and psychological distress may also consequently diminish the risk of weight gain and weight/shape concern. There should also be consideration for gender specific experiences when developing these programmes, given that the significant mediating factor varied between genders.

### Limitations

The current study provided further evidence of the impact of sexual harassment on BMI percentile and weight/shape concern on an adolescent sample and can directly influence future recommendations for studies and preventative interventions in schools. It utilised a large sample of adolescents across a range of age groups and diverse socioeconomic backgrounds. Despite the importance of these findings, there were several limitations that should be addressed. Firstly, behavioural outcomes of disordered eating pathology, such as frequency/severity of binge eating, purging or fasting behaviours, were not assessed, which could be useful to utilise in future research to determine if particular behaviours mediate this research. Secondly, more effective measures of self-objectification have since been developed and thus the measure utilised in this study may not have fully encapsulated self-objectification in both genders. Thus, our recommendation is utilising measures such as Self-Objectification Beliefs and Behaviours Scale (SOBBS) for future studies as it has been demonstrated in research to be more effective at fully encapsulating self-objectification across genders [34]. The stability of the constructs over time may have also left very little variance to be explained by other factors. Although the sample was quite big, detecting such an effect amongst control variables and highly-stable constructs may be difficult and may have contributed to the present findings. A high percentage of missing and consequent use of multiple imputation and pooled estimates means that all conclusions should be considered with caution. Finally, another limitation of this study is that BMI percentile was calculated using self-reported data and thus may be bias to recall. The study could have also benefited from another year of recruitment in order to utilise disordered eating as a mediator for BMI percentile/weight outcome in order to further investigate these relationships.

### Future directions and conclusions

Finally, this is only the second longitudinal study that has investigated the direct and indirect relationship between sexual harassment and disordered eating (BMI percentile and weight/shape concern) in an adolescent sample. Therefore, future similar studies are required to further interrogate the nature and reliability of the current findings. It is evident that preventative programmes in schools also need to target how to cope with experiences of sexual harassment, in an attempt to reduce self-objectification and psychological distress that could subsequently result in long-term disordered eating pathology and weight/shape concern.

### Strengths and limitations

The strengths of the current study are the size of the sample, its longitudinal design, its comprehensive assessment of a range of related factors using reliably validated measures and its ability to provide further evidence of the impact of sexual harassment on BMI percentile and weight/shape concern on an adolescent sample, as well as directly influencing the development of studies and preventative interventions in schools. The limitations have been discussed in depth above; however to simplify; the limitations of the current study are that some behavioural outcomes were not measured, more updated measures of self-objectification have been developed since this project started, some measures were not included at every wave, there was a high rate of missing data and the high stability of constructs over time reduces the change of explained variance by other factors, and finally BMI was self-reported. Therefore, while this current study provides vital information for future research and prevention programme development, the conclusions should be considered with caution.

### What is already known on this subject?

Prior to the current study, there was only one longitudinal study that examined the direct and indirect relationship between sexual harassment and global disordered eating pathology in an early adolescent sample after 4 years, irrespective of gender. Overall, research consistently supported the concept that sexual harassment is directly associated with disordered eating pathology and that self-objectification and psychological distress have a concurrent role in this relationship. No study to date had specifically investigated the contributing relationship of sexual harassment on weight outcomes, and weight/shape concern in adolescent population, nor potential mediators of this relationship.

### What does this study add?

The current study has provided a greater foundation to understand the role of sexual harassment on different long-term outcomes for an adolescent population; most notably the direct relationship between sexual harassment and long-term increases in self-objectification among adolescent girls. It is also directly highlights that greater preventative programmes in schools are also need to reduce sexual harassment experiences, target how to cope with experiences of sexual harassment, and attempt to reduce self-objectification and psychological distress that could subsequently result in long-term disordered eating pathology and weight/shape concern.

## Supplementary Information

Below is the link to the electronic supplementary material.

Supplementary file1 (DOCX 90 KB)

## Data Availability

The datasets generated during and/or analysed during the current study are available from the corresponding author on reasonable request.
