# Supplementary material for: Longitudinal relationship between experience of sexual harassment and 2-year body image and weight outcomes in adolescence: mediation through self-objectification and psychological distress
Source: Eat Weight Disord. 2022 Jul 14;27(8):3095–108. doi: 10.1007/s40519-022-01432-4 (PMC9803756; doi:10.1007/s40519-022-01432-4)
Supplement: Supplementary file 1 — Supplementary file1 (DOCX 90 KB) [file 40519_2022_1432_MOESM1_ESM.docx]

Longitudinal relationship between experience of sexual harassment and two-year body image and weight outcomes in adolescence: Mediation through self-objectification and psychological distress

Samantha Hayes, Alexandra Lonergan, Nora Trompeter,_,_ Kay Bussey, Phillipa Hay, & Deborah Mitchison

Corresponding Author - Miss Samantha Hayes

[Samantha.Hayes@westernsydney.edu.au](mailto:Samantha.Hayes@westernsydney.edu.au)

**SUPPLEMENTARY FILE**

**Unadjusted Mediation Models (longitudinal associations)**

*BMI Percentile*

Table 1 depicts the parallel mediation analysis for BMI percentile at Wave 3 as the dependent outcome.

*Female Participants*

In relation to Hypothesis 3, a significant mediating relationship was found through psychological distress at Wave 2 between sexual harassment at Wave 1 and higher BMI percentile at Wave 3 in female adolescents. Female adolescents who reported experiences of sexual harassment at Wave 1 reported higher levels of psychological distress at Wave 2 (a_2_=.3.92, *p* =.000), and subsequently higher psychological distress scores at Wave 2 were associated with higher BMI percentile at Wave 3 (b_2_=.34, *p* =.005). A significant association was also found between female students who reported sexual harassment at Wave 1 and higher levels of self-objectification at Wave 2 (a_1_ =-4.48, *p* =.000). However, no significant association was found between levels of self-objectification at Wave 2 and higher BMI percentile at Wave 3 (b_1_=-.05, *p* =.616). Females that reported sexual harassment did *not* report a significantly higher BMI percentile when taking into account the indirect effects of both parallel mediators (c= -.01, *p* =.726). See Figure 1 for unadjusted parallel mediation model for female adolescents. A 95% bias-corrected confidence interval based on 10,000 bootstrap samples indicated that the indirect effect through psychological distress alone (a_2_b_2_=1.34), holding other mediators constant, was entirely above zero (0.37, 2.56). Age was a significant covariate (*p* =.020).

*Male Participants*

The unadjusted parallel mediation analysis did not find a significant indirect relationship for sexual harassment and higher BMI percentile between any of the mediating variables in male adolescents. No longitudinal associations were found between experiences of sexual harassment at Wave 1 and higher self-objectification scores at Wave 2 (a_1_= -1.64, *p*=.161), self-objectification scores at Wave 2 and higher BMI percentile at Wave 3 (b_1_=.001, *p* =.885), sexual harassment at Wave 1 and higher psychological distress at Wave 2 (a_2_=1.29, *p* =.099); and psychological distress scores at Wave 2 and higher BMI percentile at Wave 3 (b_2_=.22, *p* =.262). Age was not a significant covariate (*p* =.086). Males that reported sexual harassment did *not* report a significantly higher BMI percentile when taking into account the indirect effects of both parallel mediators (c= 3.96, *p* =.201). See Figure 2 for unadjusted parallel mediation model for male adolescents.

Table 1

*Unadjusted and Adjusted Parallel Mediation Models with BMI percentile as outcome for both male and female adolescents*

|  |  | Females | | | Males | | |
| --- | --- | --- | --- | --- | --- | --- | --- |
|  |  | *B* | SE | 95%CI | *B* | SE | 95%CI |
| BMI Percentile W3 | |  |  |  |  |  |  |
|  | Age W1 | **2.25*** | **.97** | **.35, 4.16** | 2.17 | 1.25 | -.29, 4.64 |
|  | Direct effect of Sexual Harassment W1 (X) on BMI Percentile W3 (Y) | -1.58 | 2.50 | -6.48, 3.33 | 3.66 | 3.03 | -2.29, 9.61 |
|  | Path from Sexual Harassment W1 (X) to Self-Objectification W2 (M_1_) | **-4.48***** | **1.05** | **-6.55, -2.41** | -1.64 | 1.17 | -3.93, .65 |
|  | Path from Sexual Harassment W1 (X) to Psychological Distress W2 (M_2_) | **3.92***** | **.86** | **2.23, 5.62** | 1.29 | .76 | -.21, 2.79 |
|  | Path from Self-Objectification W2 (M_1_) to BMI Percentile W3 (Y) | -.05 | .10 | -.24, .14 | .001 | .13 | -.25, .25 |
|  | Path from Psychological Distress W2 (M_2_) to BMI Percentile W3 (Y) | **.34***** | **.12** | **.10, .58** | .22 | .19 | -.16, .61 |

*(continued on next page)*

|  | | Females | | | Males | | |
| --- | --- | --- | --- | --- | --- | --- | --- |
|  |  | *B* | SE | 95%CI | *B* | SE | 95%CI |
| *Mediation* | |  |  |  |  |  |  |
|  | Indirect effect of Sexual Harassment W1 (X) on BMI Percentile W3 (Y) through Self-Objectification W2 (M1), and Psychological Distress W2 (M_2_) | -.01 | 2.45 | -4.82, 4.80 | 3.96 | 3.01 | -1.96, 9.88 |
|  | Indirect effect of Sexual Harassment W1 (X) on BMI Percentile W3 (Y) through Self-Objectification W2 (M_1_) | .22 | .47 | -.68, 1.22 | -.001 | .26 | -.55,.55 |
|  | Indirect effect of Sexual Harassment W1 (X) on BMI Percentile W3 (Y) through Psychological Distress W2 (M_2_) | **1.34***_a_** | **.55** | **.37, 2.56** | .30 | .34 | -.22, 1.11 |
|  |  | *R^2^* = .01 | | | *R^2^* = .01 | | |
|  |  | *F*(2,590) = 2.85, *p* = .06 | | | *F*(2, 438) = 2.44, *p* = .09 | | |

Table 1 - *Continued*

*Note:* W1 – Wave 1; W2 – Wave 2; W3 – Wave 3. X – predictor variable; Y – Dependent Variable; M_1_, M_2_ & M_3_– Mediating variables. Unstandardized coefficients from bootstrapped analyses are presented.  * *p ≤*.05, ***p* *≤* .01, ****p* *≤*.001, _a_ CI did not include zero, indicating a significant effect.

Sexual Harassment (X)

Self-Objectification (M_1_)

BMI Percentile (Y)

Psychological Distress (M_2_)

-4.48***

3.92***

-1.58

-.05

.34***

Wave 1

Wave 2 (one year)

Wave 3 (two years)

*Figure 1*: Unadjusted parallel mediation model for sexual harassment and BMI percentile in female adolescents. Age at Wave 1 was controlled for. * *p ≤*.05, ***p* *≤* .01, ****p* *≤*.001.

Sexual Harassment (X)

Self-Objectification (M_1_)

BMI Percentile (Y)

Psychological Distress (M_2_)

-1.64

1.29

3.66

.001

.22

Wave 1

Wave 2 (one year)

Wave 3 (two years)

*Figure 2*: Unadjusted parallel mediation model for sexual harassment and BMI percentile in male adolescents. Age at Wave 1 was controlled for. **p ≤*.05, ***p* *≤* .01, ****p* *≤*.001.

*Weight/Shape Concern*

Table 2 also depicts the estimates from the parallel mediation analysis for weight/shape concern at Wave 3 as the dependent outcome.

*Female Participants*

Sexual harassment was indirectly related to weight/shape concern through a significant mediating relationship with self-objectification and psychological distress in female adolescents. A significant longitudinal association was found between sexual harassment at Wave 1 and higher self-objectification at Wave 2 (a_1_ = -4.40, *p* = .000), and between higher self-objectification at Wave 2 and greater weight/shape concern at Wave 3 (b_1_= -.03, *p* =.000). Sexual harassment at Wave 1 was also significantly associated with higher psychological distress at Wave 2 (a_2_=3.84, *p* =.000), and subsequently greater psychological distress at Wave 2 was associated with higher weight/shape concern at Wave 3 (b_2_=.06, *p* =.000) in female students. Females that had experienced sexual harassment at Wave 1 reported a significantly greater level of weight/shape concern at Wave 3 when taking into account the indirect effects of the two parallel mediators (c =.55, *p* =.000). See Figure 3 for unadjusted mediation model of this analysis.

A 95% bias-corrected confidence interval based on 10,000 bootstrap samples indicated that the indirect effect through self-objectification alone, (a_1_b_1_=.12), and psychological distress alone (a_2_b_2_=.23), holding other mediators constant, were entirely above zero (0.05 to 0.20, 0.12 to 0.36, respectively). BMI percentile at Wave 1 was a significant covariate (*p* =.000) of this relationship while age was not (*p* =.096).

*Male Participants*

Similarly, to females, no indirect relationship was found between sexual harassment and weight/shape concern through self-objectification and psychological distress in male adolescents. No longitudinal association was found between experiences of sexual harassment at Wave 1 and higher self-objectification scores at Wave 2 (a_1_= -1.60, *p*=.172), however a significant longitudinal association was found between higher scores of self-objectification at Wave 2 and greater weight/shape concern at Wave 3 (b_1_ = -.02, *p* =.001). No significant association was found between sexual harassment at Wave 1 and higher psychological distress at Wave 2 (a_2_=1.23, *p* =.121), however greater psychological distress at Wave 2 was significantly associated to higher weight/shape concern at Wave 3 (b_2_=.05, *p* =.000). Males that had experienced sexual harassment did *not* report significantly greater weight/shape concern when taking into account the indirect effects of both parallel mediators (c =.23, *p* =.067). Age and BMI percentile at baseline were significant covariates (*p* =.048, and *p*= .000, respectively). See Figure 4 for unadjusted mediation model of this analysis.

Table 2

|  | | Females | | | Males | | |
| --- | --- | --- | --- | --- | --- | --- | --- |
|  |  | *B* | SE | 95%CI | *B* | SE | 95%CI |
| Weight/Shape Concern | |  |  |  |  |  |  |
|  | Age W1 | .10 | .06 | -.02, .22 | **.10***** | **.05** | **.001, .20** |
|  | BMI percentile W1 | **.02*** | **.002** | **.01, .02** | **.01*** | **.002** | **.01, .02** |
|  | Direct effect of Sexual Harassment W1 (X) on Weight/Shape Concern W3 (Y) | .20 | .14 | -.08, .47 | .14 | .11 | -.07, .36 |
|  | Path from Sexual Harassment W1 (X) to Self-Objectification W2 (M_1_) | **-4.40*** | **1.05** | **-6.47, -2.33** | -1.60 | 1.17 | -3.89, .69 |
|  | Path from Sexual Harassment W1 (X) to Psychological Distress W2 (M_2_) | **3.84*** | **.86** | **2.15, 5.53** | 1.23 | .76 | -.28, 2.69 |
|  | Path from Self-Objectification W2 (M_1_) to Weight/Shape Concern W3 (Y) | **-.03*** | **.02** | **-.04, -.02** | **-.02*** | **.01** | **-.02, -.01** |
|  | Path from Psychological Distress W2 (M_2_) to Weight/Shape Concern W3 (Y) | **.06*** | **.01** | **.05, .07** | **.05*** | **.01** | **.04, .06** |

*Parallel Mediation Model with weight/shape concern as outcome for both male and female adolescents*

*(continued on next page)*

Table 2 - *Continued*

|  |  | Females | | | Males | | |
| --- | --- | --- | --- | --- | --- | --- | --- |
|  |  | *B* | SE | 95%CI | *B* | SE | 95%CI |
| *Mediation* | |  |  |  |  |  |  |
|  | Indirect effect of Sexual Harassment W1 (X) on Weight/Shape Concern W3 (Y) through Self-Objectification W2 (M_1_), and Psychological Distress W2 (M_2_) | **.55*** | **.15** | **.25, .85** | .22 | .12 | -.01, .45 |
|  | Indirect effect of Sexual Harassment W1 (X) on Weight/Shape Concern W3 (Y) through Self-Objectification W2 (M_1_) | **.12*_a_** | **.04** | **.05, .20** | .02 | .02 | -.01, .07 |
|  | Indirect effect of Sexual Harassment W1 (X) on Weight/Shape Concern W3 (Y) through Psychological Distress W2 (M_2_) | **.23*_a_** | **.06** | **.12, .36** | .06 | .04 | -.01, .13 |
|  |  | *R^2^* = .10 | | | *R^2^* = .11 | | |
|  |  | *F*(3 ,589) = 21.58, *p* < .001 | | | *F*(3, 437) = 17.02, *p* < .001 | | |

*Note:* W1 – Wave 1; W2 – Wave 2; W3 – Wave 3. X – predictor variable; Y – Dependent Variable; M_1_, & M_2_– Mediating variables. Unstandardized coefficients from bootstrapped analyses are presented. **p* *≤*.05, ***p* *≤* .01, *** *p ≤*.001, _a_ CI did not include zero, indicating a significant effect.

Sexual Harassment (X)

Self-Objectification (M_1_)

Shape/Weight Concern (Y)

Psychological Distress (M_2_)

-4.40***

3.84***

.20

-.03***

.06***

Wave 1

Wave 2 (one year)

Wave 3 (two years)

*Figure 3*: Unadjusted parallel mediation model for sexual harassment and weight/shape concern in female adolescents. Age at Wave 1 and BMI percentile at Wave 1 were controlled for. * *p ≤*.05, ***p* *≤* .01, ****p* *≤*.001.

Sexual Harassment (X)

Self-Objectification (M_1_)

Shape/Weight Concern (Y)

Psychological Distress (M_2_)

-1.60

1.23

.14

-.02***

.05***

Wave 1

Wave 2 (one year)

Wave 3 (two years)

*Figure 4*: Unadjusted parallel mediation model for sexual harassment and weight/shape concern in male adolescents. Age at Wave 1 and BMI percentile at Wave 1 were controlled for. **p ≤*.05, ***p* *≤* .01, ****p* *≤*.001.
